# Sleep in the wild: the importance of individual effects and environmental conditions on sleep behaviour in wild boar

**DOI:** 10.1098/rspb.2023.2115

**Published:** 2024-05-29

**Authors:** Euan Mortlock, Václav Silovský, Justine Güldenpfennig, Monika Faltusová, Astrid Olejarz, Luca Börger, Miloš Ježek, Dómhnall J. Jennings, Isabella Capellini

**Affiliations:** ^1^ School of Biological Sciences, Queen's University Belfast, 19 Chlorine Gardens, Belfast BT9 5DL, UK; ^2^ Department of Game Management and Wildlife Biology, Faculty of Forestry and Wood Sciences, Czech University of Life Sciences, Kamýcká 129, Prague 6-Suchdol 165 00, Czech Republic; ^3^ Department of Biosciences, Swansea University, Singleton Park, Swansea SA2 8PP, UK

**Keywords:** sleep ecology, biologging, double-hierarchical generalized mixed-effects models, pace-of-life syndrome, wild boar

## Abstract

Sleep serves vital physiological functions, yet how sleep in wild animals is influenced by environmental conditions is poorly understood. Here we use high-resolution biologgers to investigate sleep in wild animals over ecologically relevant time scales and quantify variability between individuals under changing conditions. We developed a robust classification for accelerometer data and measured multiple dimensions of sleep in the wild boar (*Sus scrofa*) over an annual cycle. In support of the hypothesis that environmental conditions determine thermoregulatory challenges, which regulate sleep, we show that sleep quantity, efficiency and quality are reduced on warmer days, sleep is less fragmented in longer and more humid days, while greater snow cover and rainfall promote sleep quality. Importantly, this longest and most detailed analysis of sleep in wild animals to date reveals large inter- and intra-individual variation. Specifically, short-sleepers sleep up to 46% less than long-sleepers but do not compensate for their short sleep through greater plasticity or quality, suggesting they may pay higher costs of sleep deprivation. Given the major role of sleep in health, our results suggest that global warming and the associated increase in extreme climatic events are likely to negatively impact sleep, and consequently health, in wildlife, particularly in nocturnal animals.

## 1. Introduction

Sleep is observed in virtually all animals [1]; it is essential for health, cognitive performance and development, boosting baseline immune levels and immune response to infection [2], promoting hormone production and metabolic regulation [3,4], and supporting neural maintenance, brain development and cognitive functions [5,6]. It is, therefore, not surprising that average short sleep times and sleep loss are accompanied by numerous detrimental effects on health and cognition in the short and long term [7–9], and, in the laboratory, are often followed by longer sleep which likely mitigates these costs [10]. While a wealth of evidence demonstrates its vital benefits, sleep also entails opportunity costs because sleeping animals cannot engage in fitness enhancing behaviours, like foraging or finding mates, and are exposed to greater predation risk [11]. However, sleep is believed to exhibit limited plasticity because the benefits it provides cannot be forgone for long [12]. Likewise, members of the same species are expected to have similar sleep times and patterns as their sleep is regulated by the same neurophysiological processes [12]. Whether sleep has limited variation within and between individuals is, however, unclear since it has been studied primarily in the laboratory where constraints on sleep time and benefits for reducing sleep time are absent. Furthermore, laboratory studies and the few existing sleep studies in the wild are often constrained by small sample sizes or short recording durations and consider a limited range of environmental conditions; therefore, they cannot estimate how sleep varies within and between individuals. If we are to unravel the contributions that sleep brings to the health and development of animals and quantify how the sleep benefits–costs balance differs among individuals experiencing changing conditions, we need to study sleep in the wild for long time periods. Using cutting-edge, minimally invasive biologgers, we investigated how environmental conditions influenced the quantity, efficiency, and quality of sleep within and between individuals in a wild population of wild boars (*Sus scrofa*) over the annual cycle.

Sleep time and patterns differ remarkably among species as a result of natural selection [11,13,14], but little is known about intraspecific variation in sleep and its causes and implications. While there is ample evidence that individuals within a species differ consistently and predictably in many behaviours [15], individual differences in sleep behaviour have not yet been investigated in wild animals. In humans, inter-individual differences in sleep time and patterns are well known to exist, but the magnitude of these differences is rarely quantified [16] and, importantly, it is unclear whether they reflect cultural and societal differences or natural variation [17]. Nonetheless, individuals that sleep less than average develop neurological disorders later in life, suggesting chronic short sleep durations entail long-term costs [8,9]. Similarly, whether individuals can adjust their sleep to changing conditions is poorly understood. While there is evidence of sleep loss in a handful of species, such as during large scale movement, or reproduction [18–20], previous studies are typically limited to short recording periods involving few individuals. This is problematic because wild populations experience a range of changing environmental conditions over the daily and annual cycle, and investigating these effects on sleep will permit us to quantify its plasticity over time.

In the laboratory, ambient temperature is a major driver of sleep quantity and quality [21]. As the first stage of deep sleep in mammals is characterized by low, constant body temperature, a cool ambient temperature promotes its onset, efficiency (reduced fragmentation into multiple sleep bouts), quality (longer sleep bouts) and overall daily duration [21]. Conversely, the thermoregulatory challenge presented by high temperature reduces sleep time, increases sleep fragmentation and compromises sleep quality in favour of behaviours that help thermoregulation [21,22]. As a result, ambient temperature is finely controlled in laboratory studies in humans and animals [21]. Altogether, this suggests that daily and seasonal changes in temperature should impact sleep in wild animals; accordingly, the few studies conducted under natural conditions find that cooler temperatures promote sleep [22–25]. Nevertheless, because these studies were conducted for few days/weeks, the subjects experienced only a narrow range of temperatures.

In the wild, animals are exposed to many other environmental conditions, such as humidity, rain, snow and light, that change throughout the day and across the year. These can mitigate or exacerbate the influence of temperature, but to date have been mostly neglected. For example, humidity makes thermoregulation more difficult by reducing the efficiency of evaporative cooling [26]. Thus, high humidity could compound the effects of higher temperatures on sleep, reducing duration and increasing fragmentation [21,26]. Conversely, rainfall and snow may promote sleep, by providing evaporative cooling or increasing the thermal value of bedding sites respectively.

Finally, light regulates the circadian rhythm in most mammals; hence day length controls when and how long to sleep [27]. For example, longer day lengths reduce, while shorter day lengths increase, sleep duration in humans [28]. Other light sources such as moonlight may also interfere with sleep regulation. Consistently, greater illumination from moonlight increases sleep duration in gibbons (*Hylobates* sp.) and humans, although not in baboons (*Papio anubis*) [24,25,29]. In the wild, sleep timing and duration should thus fluctuate with changing day lengths and moonlight, where longer days reduce sleep in diurnal species and increase sleep in nocturnal species. In addition, circadian type may influence the duration of sleep; for example, nocturnally active primate species sleep longer than diurnally active ones [30]. However, whether plasticity in circadian type affects sleep quantity, efficiency and quality is currently unknown. Furthermore, sleep in wild animals may be affected by human disturbance [31], particularly in nocturnally active species and by predation risk [13,32].

In this study, we investigate how changing environmental conditions influence sleep behaviour over the annual cycle in wild boar (*Sus scrofa*). Wild boar are a generalist species that exhibits considerable behavioural plasticity [33]; thus, it is a good model for investigating the plasticity of sleep under natural conditions. Studying sleep in wild animals has been difficult because of its elusive nature, and lack of reliable and minimally invasive recording equipment; thus some studies have used invasive recording equipment requiring surgery and capture–recapture methods. This approach is likely to be stressful for most wild animals and likely leads to inaccurate estimates because stress affects sleep [10,34]. Recent advances in minimally invasive biologging technology and analytical methods offer a promising solution to these problems: they allow accurate recording of behaviours and without direct observations over long time periods [35,36]. Importantly, laboratory studies with electroencephalogram on sleep in pigs, the domesticated relatives of wild boar, provide detailed information on which to base the classification of sleep with biologgers in this species [37–40].

Using multi-sensor biologgers that allow discrimination of complex behaviour in wild animals [36], we estimated total daily sleep time (TST, hours), which is an appropriate ecological estimate of sleep quantity in animals [11,13]. Moreover, unlike most previous studies in wild animals, we also considered sleep efficiency and quality to gain an ecologically meaningful and comprehensive assessment of sleep. The number of bouts over which TST occurs (sleep fragmentation/consolidation) reflects efficiency, since individuals that frequently wake up spend more time in transitional stages and less time in restorative deep sleep [11,41]. Like sleep deprivation, greater fragmentation is associated with negative effects on physiology and cognition [41,42]. Finally, the duration of the longest sleep bout in a 24 h period indicates sleep quality as deep sleep always follows lighter sleep stages in any mammal species thus far studied; hence the duration of the longest bout represents the best opportunity for an individual to accrue the benefits of deep sleep [11,41]. Consistent with this, sleep bouts following sleep deprivation are longer and deeper [10]. We also investigated to what extent individuals differ in sleep quantity, efficiency and quality and in their plasticity over time, and how these three aspects of sleep covary. We therefore asked whether individuals sleeping less exhibit greater variance (i.e. plasticity) in TST, higher sleep quality or lower sleep fragmentation that could help them mitigate the detrimental effects of an average shorter sleep time and sleep deprivation. We tested the predictions that TST is reduced, the number of sleep bouts is higher, and the duration of the longest sleep bout is shorter when ambient temperature and humidity are higher. Conversely, we expected that greater rainfall and snow depth increase TST, reduce the number of sleep bouts, and increase the duration of the longest bout. Finally, we predicted that, in a mostly nocturnally active species like wild boar, longer day length increases TST, reduces the number of sleep bouts, and increases the duration of the longest bout, while greater moonlight should increase the number of sleep bouts, reduce TST and the duration of the longest bout. Because activity period can be plastic [43] especially in generalist species [33], and nocturnally active species sleep longer [30], we account for timing of sleep within the 24 h on all three sleep measures. Wild boars are active primarily during darkness [44,45] and therefore we expect them to concentrate their sleep during daylight. Because boar also adjust their activity levels with changing environmental conditions [45,46] we predict that individuals concentrating sleep during light hours sleep longer than those that acquire some of their sleep during darkness. Lastly, we predict that TST and the duration of the longest bout decrease, while fragmentation increases, with disturbance by more human visitors [31] and the presence of wolves.

## 2. Methods

### (a) Study sites and animals

Data collection took place between 5 May 2019 and 1 December 2021 (941 days) in two areas, Kostelec (49.96 N, 14.78 E) and Doupov (50.24 N, 13.12 E), in the Czech Republic (electronic supplementary material, figure S1). Kostelec is a forested suburban area near Prague open to the public, while Doupov is characterized by mixed forest and hills, closed to the public with military and forestry access only. Wolves established a pack at Doupov in December 2020 while Kostelec has no wolves.

We employed traps to capture, immobilize, and fit 28 wild boars with customized Vertex Plus collars (Vectronic Aerospace GmbH, Berlin, Germany), carrying Daily Diaries (DD; Wildbyte Technologies Inc, Swansea, UK). Of this sample, 4 were male and 24 were female, all in reproductive age. While we have clear evidence of successful reproduction during the study period for some of these females, it is very likely that most, if not all, females reproduced since 95% of female boars older than seven months in the Czech population are reproducing [47]. The duration of recording time differed among individuals from 10 to 363 days (mean 89 days, total of 2424 days; electronic supplementary material, figure S3).

Under the same ethics permit, a captive wild boar was fitted with an identical biologging collar to those used in the wild individuals for two months. Video recordings of behaviour were collected so that the data derived from the biologger could be validated against observed behaviours. This animal was housed in a wildlife enclosure (approx 1 km^2^) covered by mixed forest in Bohumile (Czech Republic), owned and managed by the CZU.

### (b) Classification of sleep

The Daily Diary (DD) [36] is an inertial measurement unit (IMU) containing multiple sensors, including tri-axial accelerometers that capture acceleration data quantified in units of ‘*g*’ (1*g* = 9.81 m s^−2^) across a range of ±16*g* and varying in frequency from low (10 Hz) to high (100 Hz), as set by the user (here set to 20 Hz). This allows DDs to detect a range of movement and behaviour, from subtle to vigorous, as acceleration is measured in three dimensions [48]. When the device is at rest, such as when an animal is asleep, the total force exerted on all three axes sums to approximately 1*g* (the effect of gravity), thus allowing the inference of posture based on the known, calibrated orientation of the device. While accelerometers and other biologging sensors are growing more popular generally [35], and can be used to study sleep in the wild [49], a naive application of metrics derived from biologgers risks conflating wakeful rest and sleep. Thus, we built a robust classification of sleep with DD based on laboratory studies in domestic pigs that provide a detailed description of sleep postures and sleep estimates quantified with electroencephalogram.

**Figure 1.**
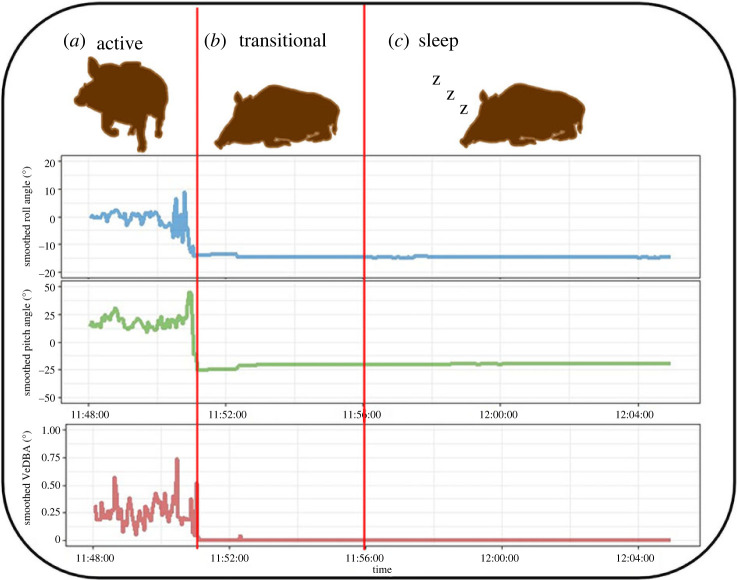
Example of DD data showing changes in smoothed roll, pitch and VeDBA values, corresponding to relevant behavioural types (separated by vertical red lines), to identify the onset of sleep. (*a*) General active behaviours such as movement and foraging; (*b*) sternal recumbency for the period of drowsiness/transitional stage; and (*c*) an individual is classified as asleep if it remains in sternal recumbency for longer than 5 min with little to no movement.

During sleep individuals enter a state of quiescence in a species-specific posture and require a stronger stimulus to elicit a response compared to awake individuals [1] but, unlike torpor and hibernation, return rapidly to a waking state [1]. Pigs sleep in either lateral or sternal recumbency with the head on the ground [38–40], accompanied by rapid loss of muscle tone at sleep onset [38]. Posture was derived from raw acceleration by calculating static acceleration; this isolates the gravitational component from dynamic acceleration (acceleration resulting from movement alone). Static acceleration was estimated using a 2 s running mean filter [50] applied to the raw 20 Hz data. Body pitch and roll angles were then derived from static acceleration using the arcsine of the surge (forward–backward) and sway (left–right) axis [48]. Sternal recumbency with head-down was defined as (*pitch* < 0°) and (*roll* > −15° and <+15°), while lateral recumbency was defined as (*roll* < −15° and >+15°). Sustained lack of movement, the other key behavioural cue for sleep, was identified using the dynamic acceleration (i.e. produced by movement only). This was calculated by subtracting static acceleration from raw acceleration on each axis (*Ax*, *Ay*, *Az*). We used a metric combining dynamic acceleration on all three axes, vectorial dynamic body acceleration (VeDBA), calculated as

$$\mathrm{VeDBA}=\;\sqrt{A_{x}^{2}+\;A_{y}^{2}+A_{z}^{2}}\;,$$

where $A_{x}^{2}$, $A_{y}^{2}$, $A_{z}^{2}$ are the dynamic components of each of the three axes of acceleration [51]. Smoothed VeDBA was then calculated over a 2 s smoothing window of raw VeDBA (20 Hz). Employing VeDBA allowed us to distinguish between the animal's active and inactive states at a high resolution. We set a threshold for movement in sleep postures to 0.2 VeDBA such that sleep bouts ended if this threshold was crossed but allowed for minor movements, such as those due to postural adjustment during sleep. Finally, given that domestic pigs in recumbent posture require 4–5 min to transition from wakefulness to sleep [37,38], we discarded the initial 5 min of each sleep bout. Our classification thus distinguishes sleep from wakeful rest using behavioural markers for sleep (figure 1; electronic supplementary material, figure S4).

To assess the accuracy of our posture classification, behavioural observations of the captive boar were collected opportunistically with a handheld camera (Sony HDR-CX625). Footage of the individual in lateral and sternal recumbency was time-matched to the acceleration data. As our criteria for identification of sleep bouts filtered out any periods shorter than 5 min, we adjusted the time threshold to the duration of the shortest bout of recumbency in these videos (i.e. 31 s), allowing shorter periods to be included in accuracy testing. In total, 38.8 min of video were used, of which 11.4 min of recumbent postures and 27.4 min of other behaviours. Accuracy metrics were scored at 1 s resolution. Our classification distinguished lateral and sternal recumbency with an accuracy of 98% and specificity of 97% (electronic supplementary material, table S1).

From the biologger data we estimated the following sleep parameters for every 24 h: TST (hours); number of sleep bouts; duration of the longest sleep bout (minutes). In addition, following a commonly used approach to categorize diurnality, nocturnality and cathemerality in animal behaviour [43], we calculated the distribution of sleep (DS) as percentage of time slept between sunrise and sunset out of total daily sleep time. We discarded the first and last day of recording for each animal as these were incomplete 24 h periods.

### (c) Environmental data

Hourly weather data were taken from the Jevany (Kostelec, 49.96 N, 14.80 E) and Kyelska Spa (Doupov, 50.26 N, 13.02 E) weather stations (www.visualcrossing.com). Daily means were computed for ambient temperature (°C); snow depth (cm); and relative humidity (the amount of water vapour present in the air compared to the maximum amount possible for a given temperature, as a percentage, %). Precipitation (mm) was quantified as the total daily rainfall. Day length (hours) was estimated as hours of light from sunrise to sunset. Moon phase was coded as a continuous variable ranging from new moon (dark; 0) to full moon (bright; 1). Based on [44], we adjusted the values for moon phase to account for cloud cover that reduces night brightness. We thus calculated a ‘cloud-adjusted moon phase’ by computing the percentage cloud cover as a percentage of the moon phase, and subtracting this from moon phase value. Electronic supplementary material, table S2, reports the range of environmental conditions recorded over the study period. We controlled for the potential impact of wolves on sleep with a binary predictor. We coded as ‘wolf presence’ at Doupov from December 2020 onwards, and coded Doupov prior to this date and Kostolec as ‘wolf absence’. We controlled for the possible influence of human presence on sleep [31] by including as a fixed effect the total daily number of visitors recorded with an automated counter (Mobile multi, Eco-Compteur) [52] at Kostelec. The number of visitors at Doupov was coded 0, as this area is closed to the public.

### (d) Statistical analysis

We used double-hierarchical generalized linear mixed-effects models (DHGLMs) to assess how wild boar altered their sleep in relation to changing environmental conditions [53]. Specifically, we modelled the changes in the mean of the three sleep measures (TST, number of sleep bouts, duration of the longest sleep bout) and their variance (sigma (*σ*) component) in a Bayesian framework with the R package ‘brms’ [54,55], and the Stan open source modelling platform [56]. Unlike standard linear mixed-effects models, DHGLMs can handle non-heterogeneous residual errors, allowing a more accurate assessment of fixed effects when the duration of recording time differs between individuals, as in our study (electronic supplementary material, figure S2) [57].

Prior to analyses, the duration of the longest sleep bout was log-transformed and all fixed effects were centred and scaled to improve model fitting. We assigned Gaussian distributions to response variables. Our data were hierarchically structured, thereby measures of sleep were nested inside individual ID. Therefore, we included ID as a random effect for both the mean and variance (sigma) component of each model to determine inter- and intra-individual variation in the three sleep measures, i.e. differences between individuals in sleep characteristics and in their plasticity, respectively. Thus, for each sleep parameter this model structure allows us to quantify the population mean and variance over the study period through the intercept (mean) and variance (sigma) fixed effects, and to quantify inter-individual differences in mean and variance (i.e. individual plasticity) through the intercept and variance (sigma) of the ID random effects respectively. We also estimated the correlation between the mean and variance of the random effect ID to investigate whether individuals with higher mean value of the sleep parameters also exhibited greater or lower variance (i.e. plasticity) in that sleep parameter. Finally, we included an autoregression term of order 1, applied to each individual, to control for temporal autocorrelation.

Environmental conditions, human visitation, wolf presence, DS, area, sex and year were treated as fixed effects, and we included month of the year to account for seasonal changes not captured by environmental predictors. We used a second-order polynomial term to capture possible nonlinear effects of high and low temperatures, and the distribution of sleep across 24 h, on sleep. Further, because the effect of ‘month’ is cyclical (e.g. where month 12 is more similar to month 1 than month 6), we used a second-order polynomial term applied both as a fixed effect and a random slope term in the model formula. Widely applicable information criterion (WAIC) confirmed that models fitted with the random slope for month provided a better fit to the data than an intercept-only model (ΔWAIC > 7 indicates a superior model fit).

We ran models using Markov chain Monte Carlo (MCMC) with weakly informative, normally distributed priors for the fixed effects with a mean of 0 and variance of 100. We assigned weakly informative, scaled *t*-distributed priors with 3 degrees of freedom to the random effects (individual-level variation) and error terms in both components of the models [58]. We ran chains of 15 000 iterations with a burn-in of 1000 iterations for the three models, sampling every 15th iteration. Visual inspection of the traces in the resulting posterior distributions showed adequate mixing and convergence. The Gelman–Rubin convergence statistic (Rhat) confirmed convergence as values were equal to 1 for all parameters [59]. Effective sample size (ESS) for all estimated parameters over 1000 confirmed that the posterior distributions had negligible levels of autocorrelation (electronic supplementary material, tables S2–S4). Models were run in triplicate and converged on similar solutions.

All fixed effects were first entered simultaneously in a starting maximal model. We reduced the maximal model by eliminating the least meaningful fixed effect, starting from quadratic terms if not meaningful, and re-ran the model, repeating the procedure until only meaningful predictors remained in a minimal statistically justifiable model (reduced model [50]). Fixed effects predictors and the correlation between mean and variance of the ID random effects were considered meaningful if the percentage of the posterior distribution crossing zero in the opposite direction of effect was less than 5 (percentage across-zero: *P*_x_ [49]). In the DHGLM, random effects estimates are constrained to be greater than zero as they are calculated on a log scale, making *P_x_* unsuitable for assessment of effects. Therefore for random effects we considered a posterior distribution shifted away from zero, and the 95% CI not abutting zero, as evidence for a meaningful effect. Finally, we investigated whether, at the population and individual level, a greater TST also resulted in more sleep bouts per day and increased duration of the longest bout per day. To this end, we ran two mixed-effects models in the same Bayesian modelling framework, with TST as the response and number of bouts per day or duration of the longest bout per day as predictor, using normally distributed priors with a mean of zero and variance of 10 000. We included a random slope (ID slope) for the predictor with a random effect for individual (ID intercept), an intercept-only random effect for year, and an autocorrelation structure of order 1 applied at the individual level. The model also estimated correlations between individual random slope (ID slope) and individual intercept (ID intercept) at the population level, to investigate whether individuals with a higher or lower mean TST showed a bigger or smaller change in TST with bouts per day or duration of the longest bout per day.

## 3. Results

For each model, we report below the median and credible intervals of the posterior distributions of meaningful effects within brackets; results for the maximal and reduced models are in the electronic supplementary material, tables S3–S8; S10–S11.

### (a) Sleep quantity: total sleep time (hours per day)

**Figure 2.**
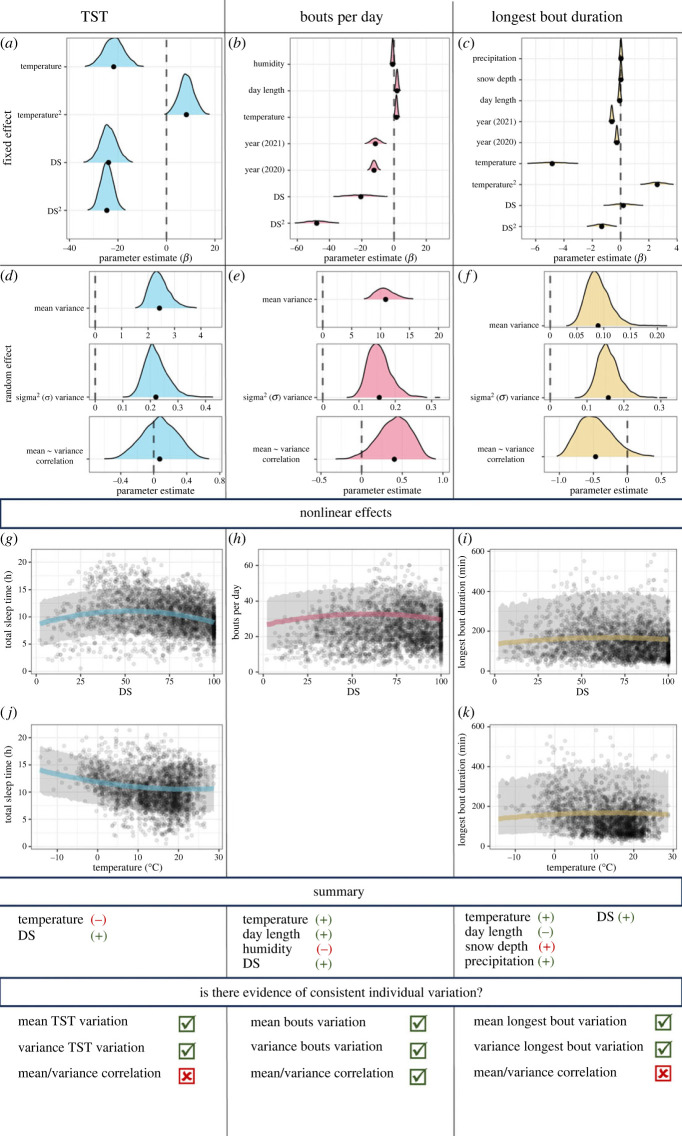
Reduced model results for total sleep time (TST, blue), number of bouts per day (red) and duration of the longest sleep bout per day (yellow). (*a–c*) The posterior distributions for the fixed effects, with dashed line denoting 0. (*d–f*) Posterior distributions of the random effects for inter-individual differences in the mean (ID intercept) and variance (ID sigma) of the sleep parameter and their correlation (ID intercept approx. ID sigma). (*g*–*k*) Nonlinear predicted effects of the distribution of sleep over 24 h (DS) and temperature. The summary reports the direction of effect for environmental variables and the general direction of effect for nonlinear DS (denoted with ±), and evidence of individual variation from the individual effects.

Boars slept primarily during daylight, although some individuals slept also during darkness (electronic supplementary material, figure S5), and there were no days of total sleep deprivation (electronic supplementary material, figure S5). From a maximal model with all predictors (electronic supplementary material, table S6), the reduced model found a quadratic effect for both temperature (temperature: −21.73 [−29.81, −19.67]; temperature^2^: 8.17 [2.11, 14.31]; figure 2*a*,*j*; electronic supplementary material, table S3) and the distribution of sleep over 24 h (DS: −24.02 [−30.43, −17.04]; DS^2^: −24.61 [−29.40, −19.67]; figure 2*a*,*g*; electronic supplementary material, table S3). Thus, TST was shorter when some sleep occurred during darkness and at higher temperatures (figure 2*g*,*j*). The model estimated that boars slept for 10.43 h per day across the year, ranging from an average of 10.14 h in summer (June–August) and an average of 12.22 h in winter (November–February). However, the random effects for between-individual variation revealed differences in their mean TST (2.39 [1.84, 3.29]; electronic supplementary material, table S3; figure 2*d*), as the model estimates varied from 6.81 h for the shortest sleeping individual to 14.94 h for the longest sleeping individual (electronic supplementary material, table S9). Individual boars also differed in the variance in TST (0.21 [0.15, 0.31]; electronic supplementary material, table S3; figure 2*d*), with estimates ranging from 1.09 to 3.20 h across individuals (electronic supplementary material, table S9). These values thus ranged across individuals from a minimum 12.86% to a maximum of 37.39% of TST (electronic supplementary material, table S9). There was no correlation between mean TST and variance in TST at the individual level (0.08 [−0.34, 0.47]; electronic supplementary material, table S3; figure 2*d*), indicating that short-sleepers did not exhibit greater or lower variance in TST than long-sleepers.

### (b) Sleep efficiency: number of sleep bouts per day (sleep fragmentation/consolidation)

From a maximal model with all predictors (electronic supplementary material, table S7), the reduced model identified a curvilinear effect of the distribution of sleep over 24 h (DS: −20.49 [−36.21, −4.65]; DS^2^: −47.98 [−60.45, −35.25]; figure 2*b*,*h*; electronic supplementary material, table S4). The model also indicated that the number of sleep bouts increased linearly with warmer temperature (1.54 [0.74, 2.38]) and longer days (1.92 [1.02, 2.79]), but decreased with greater humidity (−0.85 [−1.27, −0.41]; figure 2*b*; electronic supplementary material, table S4). Finally, sleep was less fragmented in 2020 (−12.40 [−15.08, −9.64]) and 2021 (−11.56 [−16.69, −6.52]; figure 2*b*; electronic supplementary material, table S4) compared to 2019.

The model estimated that, on average, boars slept in 24.44 bouts per day across the year, ranging from a minimum of 17.49 in winter to a maximum of 25.21 in summer. The random effects revealed that individuals differed in the mean number of sleep bouts (10.67 [7.97, 15.02]; electronic supplementary material, table S4; figure 2*e*), which ranged across individuals from a minimum of 13.58 bouts per day for the boar sleeping more efficiently to a maximum 35.27 bouts per day for the individual exhibiting the greatest sleep fragmentation. Model estimates for individual boar also differed in the variance in the number of sleep bouts (0.15 [0.10, 0.23]; electronic supplementary material, table S4; figure 2*e*), with model estimates varying from 4.56 to 13.91 bouts per day across individuals. There was a positive correlation between mean number of sleep bouts per day and variance in the number of sleep bouts per day at the individual level (0.42 [−0.01, 0.74]; electronic supplementary material, table S4; figure 2*e*), indicating that boar sleeping in more bouts also exhibited higher variance in the number of bouts.

### (c) Sleep quality: duration of the longest sleep bout

From a maximal model with all predictors (electronic supplementary material, table S8), the reduced model for duration of the longest bout identified a weak curvilinear effects of temperature (temperature: −4.84 [−6.67, −3.03]; temperature^2^: 2.59 [1.58, 3.59]; electronic supplementary material, table S5; figure 2*c*,*i*) and the distribution of sleep over 24 h (DS: 0.20 [−1.11, 1.49]; DS^2^: −1.33 [−2.25, −0.41]; electronic supplementary material, table S5; figure 2*c*,*k*) such that the longest bout was shorter when some sleep occurred during darkness and lower temperatures. The model also found that the duration of the longest bout was shorter with longer day length (−0.06 [−0.10, −0.03]) but increased with greater snow depth (0.02 [0.00, 0.04]) and precipitation (0.03 [0.01, 0.04]; electronic supplementary material, table S5; figure 2*c*). Finally, the duration of the longest bout was shorter in 2020 (−0.26 [−0.35, −0.18]) and 2021 (−0.62 [−0.70, −0.51]) than 2019, and in 2021 compared to 2020 (electronic supplementary material, table S5; figure 2*c*).

The model estimated that, on average, the longest sleep bout lasted for 2.10 h across the year, ranging from a minimum of 1.99 in summer and a maximum of 2.83 in winter. The random effects of the model showed that individual boar differed in the estimated mean duration of the longest sleep bout (0.09 [0.05, 0.14]; electronic supplementary material, table S5; figure 2*f*) and in the variance of the longest bout duration (0.16 [0.11, 0.23]), with model estimates ranging from 1.36 to 1.81 h per day across individuals (electronic supplementary material, table S5; figure 2*f*). Finally, there was a positive correlation between mean duration and variance in the longest bout at individual level (−0.49 [−0.87, 0.09]; electronic supplementary material, table S5; figure 2*f*), indicating that boars with greater duration for the longest sleep bout exhibited higher variance in its duration.

### (d) Relationships between sleep parameters

At the population level, TST and the number of bouts per day were unrelated (0.02 [−0.02, 0.05]; electronic supplementary material, table S10); thus, TST did not increase with more sleeping bouts but individuals varied in their slope between TST and the number of bouts per day (0.08 [0.05, 0.11]; electronic supplementary material, table S10). Furthermore, there was a negative correlation between the random intercept (ID intercept) and random slope (ID slope) for number of bouts per day, indicating that boars with greater TST were more likely to have a negative relationship between mean TST and number of bouts per day, while those with lower TST were more likely to show a positive relationship (−0.73 [−0.88, −0.44]; electronic supplementary material, table S10, figure S7*a*).

At the population level, greater TST was associated with increased duration of longest bout per day (0.68 [0.36, 1.03]; electronic supplementary material, table S11), hence when the duration of the longest bout increased so did total daily sleep time. Individuals varied in this relationship with some showing a greater slope for TST with duration of longest bout than others (0.67 [0.39, 1.03]; electronic supplementary material, table S11, figure S7*b*). There was also a strong negative correlation between mean TST and the slope between TST and the duration of the longest bout, indicating that individuals with a lower mean TST showed a stronger increase in TST as sleep quality increased (−0.77 [−0.91, −0.37]; electronic supplementary material, table S11).

## 4. Discussion

Sleep is vital, yet how it varies within and between individuals, and responds to changing environmental conditions over time remains an open question. Recent studies have started to use minimally invasive, accelerometer-based measurements of sleep in wild animals to improve understanding of the ecology of sleep [25]. Here, in the longest and most detailed analysis of sleep in wild animals to date, we show that boars slept on average for 10.43 h per day in 24.40 bouts, with the longest bout lasting on average 2.10 h, but, contrary to expectations [12] individual wild boars differed markedly in the mean sleep quantity (TST), efficiency (sleep fragmentation/consolidation as the number of sleep bouts per day), quality (duration of the longest bout per day), and in their variance, i.e. plasticity. Sleep responded plastically to changes in environmental conditions that influence thermoregulation. Specifically, total daily sleep time was approximately 17% shorter, more fragmented, and of poorer quality in hot, summer days, potentially leading to sleep deprivation. Conversely, rainfall and snow favoured higher sleep quality. Thus, contrary to the idea that sleep is inflexible and consistent between individuals [12], we demonstrate that sleep differs between individuals and responds plastically and predictably to changing environmental conditions. Since sleep is an essential self-maintenance process, these results suggest that individuals differ in the amount of benefit gained from sleeping, and in their ability to mitigate the costs associated with sleep deprivation.

Our analytic approach reveals a complex diversity in sleep between and within individuals that has not been previously quantified in nonhuman animals and highlights the importance of considering multiple dimensions of sleep. We expected that individuals consistently sleeping less could try and mitigate the costs of their average shorter sleep through greater plasticity (i.e. variance) in TST, higher sleep quality (longest bout duration) and/or greater efficiency (fewer sleep bouts per day). We find that boars differ in sleep quantity (TST individual mean) and its plasticity (TST individual variance), with short sleepers sleeping up to 46% less per day than long sleepers. Contrary to our expectations, short-sleeping individuals do not show greater plasticity in sleep quantity relative to long-sleeping individuals, nor do they enjoy higher sleep quality given that sleep quantity and quality increase with one another. Therefore, short-sleeping individuals do not compensate for their short sleep by increased plasticity or quality. Boar also differ in sleep fragmentation; individuals sleeping less efficiently distribute sleep in over twice as many bouts as those sleeping more efficiently and show higher plasticity in fragmentation suggesting that they likely attempt to compensate their poorer sleep by increasing the number of bouts. However, sleeping in more bouts does not systematically lead to longer TST, as sleep quantity and fragmentation are unrelated. Specifically, we find that only few individuals can increase TST by sleeping in more bouts, others reduce TST with more bouts presumably by increasing sleep depth, while in most boar TST does not vary with the number of sleep bouts (electronic supplementary material, figure S7). Considering that wild baboons fail to exhibit compensatory sleep rebound after a poor night's sleep [25], altogether our results suggest that individuals sleeping consistently less, or less efficiently, are likely to gain fewer benefits of sleep and may pay higher costs when sleep deprived.

We propose that the plasticity of sleep may vary across species in relation to their ecology. Our findings show that sleep in wild boar aligns with the general behavioural flexibility and adaptability documented for this species [33,60,61]. We thus expect similar levels of sleep inter- and intra-individual variation in cathemeral species, longer-sleeping species such as in many predators [11], and those exhibiting greater behavioural plasticity. However, species that sleep little in the laboratory, such as artiodactyls and perissodactyls [13,38], are likely at the physiological minimum for sleep in mammals, and may show lower inter- and intra-individual differences, and plasticity. Quantifying sleep variation within and across species will help evaluate to what extent globally increasing environmental changes, such as land change use, climate change and other anthropogenic stressors, impact on the key benefits that wild animals accrue from sleeping. Estimating time spent in each of the two mammalian sleep stages, REM (rapid-eye-movement) and NREM (non-REM) sleep, will permit improved investigation of costs and benefits of each sleep stage [13,30]. However, distinguishing REM and NREM sleep is currently an intractable problem for long-term, minimally invasive monitoring of wildlife. High-resolution accelerometery represents a promising step towards this goal, as shown in this study, and future developments in sensor technology and analysis might help detect physiological parameters associated with sleep stages (e.g. body temperature, heart and breathing rate).

Ambient temperature and light affect sleep quantity, fragmentation and quality in the laboratory [27,62]; thus, they are considered important mediators of sleep behaviour. However, how multiple environmental conditions influence sleep in the wild has not been investigated. Weather and light levels are also known to influence activity patterns in wild boar; for example, higher temperatures during the night and low moonlight favour nocturnal activity [44,45,63]. Likewise, our study reveals that the broad range of environmental conditions that wild animals face over time also affects sleep in more complex ways than anticipated. Consistent with the hypothesis that higher temperatures impair sleep [21,26], we find that sleep is shorter, more fragmented and of lower quality at higher temperature. Unexpectedly, though, higher humidity leads to greater sleep consolidation into fewer bouts. Conversely, precipitation and snow which are expected to favour thermoregulation and sleep, increase sleep quality. Contrary to predictions that light promotes sleep in nocturnal species, sleep in wild boar is more fragmented and of lower quality during longer days, but unaffected by our cloud-adjusted measure of moon phase. Therefore, winter favours sleep since lower temperature and snow likely enhance the thermal value of bed sites [62,64], and snow increases the energetic cost of travelling [46]. Conversely, sleep is shorter, fragmented and of poorer quality in summer which is the reproductive season for temperate species. Since female mammals with dependent offspring trade off sleep for parental care in captivity [18,20], our results suggest that hot and humid summer days may exacerbate the detrimental effects of sleep loss in reproducing females. Reproduction and thermoregulation may also help explain why individuals that sleep more during the night sleep less overall. Because piglets sleep in short, frequent bouts [40] and are likely more challenged by high temperatures than adults [65], we suggest that lactating females might shift sleep during darkness in response to offspring needs. Consistent with this suggestion, sleeping patterns and circadian rhythm of neonates are still developing during early life [66], and during the farrowing season female boars increase diurnal activity leading to a polyphasic activity pattern [67]. Given the vital role of sleep for health [4], we propose that compromised sleep in reproducing individuals likely contributes to explaining some costs of reproduction, such as compromised immunity, lower survival and reduced future reproductive investment [68,69].

We did not detect any influence of the arrival of wolves and human visitation on sleep in wild boar. However, this is likely due to the fact that changes in sleep due to predation risk or human disturbance occur are brief and occur at very short temporal scales [31,32]. Thus, to assess whether predation risk affects sleep in wild animals, targeted studies with high resolution data at short temporal scales are needed, for example with data on where and when predators move relative to prey, and where and when predation events occur. Likewise, behavioural responses to short-lived changes in human activity are difficult to detect and are often context-dependent, thus only fine-scale approaches can reveal these effects [31,70].

Given the essential benefits of sleep [2], we propose that sleep can be viewed as a behaviour favouring self-maintenance and survival; furthermore, the variation among individuals may be explained by models such as the extended pace-of-life syndrome theory (ePOLS) [10,71]. According to ePOLS, individual differences in behavioural and physiological traits covary with life-history traits. Therefore, ‘fast-living’ individuals are expected to grow quickly, invest more in reproduction and less in self-maintenance, and ultimately die younger. At the opposite extreme, ‘slow-living’ individuals should grow slowly, invest more resources in self-maintenance than reproduction, and live longer. Thus, shorter sleep may represent a facet of the ‘fast’ living strategy where sleep is reduced in favour of behaviours that enhance reproductive investment. Consistent with this suggestion, individual differences in TST in fruit flies (*Drosophila melanogaster*) are genetically determined and shorter-sleeping flies die younger [72]. Given that sleep loss comes at substantial costs [10], we expect that individuals with shorter and more fragmented TST exhibit traits such as reduced immunocompetence, and may be particularly affected by sleep deprivation, for example impaired cognitive abilities like decision making (e.g. response time to approaching predators, navigation). We thus anticipate that short-sleeping individuals within species show correlated tendencies with traits such as growth rate and reproductive behaviour.

To conclude, our study further demonstrates that high-resolution biologgers can be used to investigate sleep in wild animals [49] over ecologically relevant time scales. This approach reveals meaningful and unpredicted inter- and intra-individual differences in daily sleep quantity and efficiency that likely have important implications for health, cognitive abilities, and response to natural and anthropogenic stressors in wildlife. In contrast to views that sleep has limited plasticity [11,73], our study demonstrates that sleep in the wild is shaped by changing environmental conditions that affect thermoregulation in more complex ways than anticipated. Importantly, sleep is shorter, more fragmented and of lower quality in warmer temperatures, leading to a reduction of sleep time by 17% in summer. Given the major role that sleep plays in health [2,41], global warming and the associated increase in extreme climatic events are likely to impact sleep, and consequently health, in wildlife, particularly in nocturnal animals in unpredictable ways. Such detrimental effects may be further exacerbated if wild animals are exposed to anthropogenic stressors that disrupt sleep.

## Data Availability

Supplementary material is available online [74].
